# Control of Disabling Vertigo in Ménière’s Disease Following Cochlear Implantation without Labyrinthectomy

**DOI:** 10.3390/audiolres12040040

**Published:** 2022-07-22

**Authors:** Andrea Canale, Giulia Dalmasso, Roberto Albera, Sergio Lucisano, George Dumas, Flavio Perottino, Andrea Albera

**Affiliations:** 1 Department of Surgical Sciences, University of Turin, 10124 Torino, Italy; andrea.canale@unito.it (A.C.); roberto.albera@unito.it (R.A.); 2 Service Otorhinolaryngologie, Centre Hospitalier des Escartons, 05100 Briançon, France; giulia.dalmasso89@gmail.com (G.D.); fperottino@ch-briancon.fr (F.P.); 3 Otorinolaringoiatria U, Città della Salute e della Scienza di Torino, 10126 Turin, Italy; slucisano@cittadellasalute.to.it; 4 Service Otorhinolaryngologie, Centre Hospitalier Universitaire Grenoble Alpes, 38100 Grenoble, France; georges.dumas10@outlook.fr

**Keywords:** Ménière’s disease, cochlear implant, vertigo, vestibular system, hydrops

## Abstract

Background: The placement of a cochlear implant (CI) can restore auditory function in the case of profound cochlear deafness, which may be due to Ménière’s disease (MD) or be associated with symptoms related to endolymphatic hydrops. The usual treatment of disabling vertigo in MD is based on vestibular deafferentation by labyrinth ablation. The aim of the present study was to retrospectively evaluate the efficacy of the CI in the control of disabling vestibular manifestations in the case of MD unresponsive to medical treatments. Methods: A case series of five MD patients with disabling vestibular manifestations associated with profound hearing loss was included. A complete audio-vestibular evaluation was performed after CI positioning. Results: All patients reported clinical benefits after implant positioning: no vestibular crisis was reported after the surgery. The vHIT and the caloric test showed a normal function or a mild vestibular hypofunction. The auditory performances were comparable to those in the general implanted population. All patients reported subjective tinnitus reduction. Conclusions: To date, very few studies have reported vestibular outcomes in hydropic pathology on the implanted side; our results are encouraging. We can therefore confirm the efficacy and safety of the CI as a unique treatment for hearing loss, dizziness, and tinnitus in case of disabling cochlear hydrops, especially in those patients where the history of the disease requires preservation of the vestibular function.

## 1. Introduction

Ménière’s disease (MD) is a chronic disease of the inner ear, which, according to the revised guidelines proposed by the Bárány Society in 2015, is “defined” as the occurrence of two or more episodes of objective vertigo lasting more than 20 min, and documented low- to medium frequency sensorineural hearing loss, auricular fullness, and tinnitus [1]. The pathophysiology of MD was initially proposed by Schuknecht in 1975 as a blockage of the endolymphatic duct causing hydrops, with the rupture of the distended Reissner’s membrane and consequent vertigo due to potassium contaminating the perilymph around the afferent nerve fibers [2]. Recently, an alternative theory has been proposed by Gibson, involving a sudden increase of endolymphatic volume causing a shift in fluid from the pars inferior (cochlea) to the pars superior (utricle and semicircular canals), and stretching the vestibular hair cells within the cristae of the semicircular canals [3].

In addition to the well-known progressive hearing loss, the effects of hydrops on the vestibular system are often disabling; the impact of the crisis on everyday life can be classified by the “functional level scale” proposed by the American Academy of Otolaryngology and Head and Neck Surgery (AAO-HNS) in 1995, considering the disease as disabling from stage 4 [4]. This scale can also be useful to justify a treatment and to verify its effectiveness; an audio-vestibular evaluation is recommended before each treatment, including at least: pure-tone audiometry, determination of speech recognition threshold (SRT), vestibular evoked myogenic potentials (VEMPs), video head impulse test (vHIT), distortion product otoacoustic emissions (DPOAEs), and an electrocochleography [5]. Subjective evaluation of the impact of dizziness can be assessed through questionnaires, such as the Dizziness Handicap Inventory (DHI) [6] and the Ménière’s Disease Patient-Oriented Severity Index (MDPOSI) [7]. The first line of treatment for MD involves improving the general wellness and habits of the patients, with regular hydration, low-stress levels, and taking care for the quality of sleep. Betahistine, diuretics, and intratympanic steroid injections are some of the medical conservative treatments for the disease. In the event that conservative treatments have proved ineffective, and dizziness persists disabling, selective ablation of the peripheral vestibular system by means of intratympanic injections of Gentamicin, vestibular neurotomy, or surgical labyrinthectomy may be considered [8]. Bilateral MD and unilateral MD (an extension of the surgical indication to implant in unilateral deafness) are excellent conditions for applying a cochlear implant (CI) to restore auditory functioning. The few results found in the literature are varied in terms of hearing; the series taken into consideration is always quite small. A recent study on this topic, considering 27 patients, showed better hearing results in MD cases compared to the general implanted population [9]. In these patients, the CI can be placed with, without, or after the labyrinthectomy. In case of bilateral disease, lateralization of the disease on the previous labyrinthectomy, or in case of an unidentifiable side of the disease, vestibular destructive procedures cannot be performed due to the risk of causing a complete lack of vestibular afferents. In this study, we present a case series of five disabling definite MD or disabling delayed hydropic manifestations with concomitant profound hearing loss that were exclusively treated with CI. The literature suggests performing simultaneous labyrinthectomy and cochlear implantations in patients with end-stage MD as it ensures the resolution of vertigo and can yield good hearing outcomes and improvements in sound localization, speech understanding, and tinnitus severity [10]. However, we were forced to this surgical indication of cochlear implantation without labyrinthectomy to avoid the risk of chronic unsteadiness as the patients already had contralateral vestibular deficits or we were not aware of the side that was affected by the disease. The surprisingly excellent results obtained in terms of vertigo control after cochlear implantation were retrospectively investigated in the present study by analyzing the vestibular function with objective tests and with the assessment of the subjective perception of the quality of life.

## 2. Materials and Methods

Five patients suffering from disabling vertigo crisis due to MD, unresponsive to medical treatment, or delayed endolymphatic hydrops (DEH), were submitted to unilateral CI positioning without surgical labyrinthectomy. The cochlear electrodes of all patients included in the study were inserted with a classic approach through the round window. No noteworthy intraoperative or postoperative surgical complications were reported.

Each patient was postoperatively tested with a series of evaluations at an average time of 22 months after CI activation (range 4 months–6 years): Romberg’s test, 

Fukuda (Unterberger) test, caloric test, vHIT, pure-tone audiometry, and speech audiometry with disyllables in free field and quiet conditions. Furthermore, each patient completed two different questionnaires, the Dizziness Handicap Inventory (DHI) and the Short Form Quality of Life Questionnaire (SF36-QOL Italian version) [11]. Both questionnaires and their scorings are reported in the appendix. As for the DHI, a maximum of 100 points indicates the greatest disturbance to the patient and a minimum of 0 points suggests that there is no handicap. Conversely, a maximum score of 100 on SF36-QOL is achieved only when subjects rate their health very favorably.

Before undergoing surgery, each patient was submitted to pure-tone audiometry, a CT scan of the temporal bone, and labyrinthine magnetic resonance imaging (MRI). Both preoperative and postoperative hearing thresholds mentioned in this paper refer only to the pure-tone average (PTA—mean hearing threshold at 0.5–1–2–4 kHz). The postoperative outcomes of the vHIT and the DHI were compared with those exams performed preoperatively; contrarily, due to the retrospective nature of the study, and since the execution of the caloric test is not mandatory to diagnose MD, the preoperative results of the caloric test were not present. The follow-up continues to date, with an average of 29 months (range from 13 months to 8 years).

The vHIT was performed following a standard procedure [12], which requires at least 20 valid head impulses with peak velocity ranging from 100 to 300°/s for each semicircular canal, according to the planes of stimulation (Natus Medical Incorporated, Pleasanton, CA, USA). Each participant wore a lightweight goggle frame with a built-in infrared camera to record right eye movements and an accelerometer to record head movements at a sampling frequency of 250 Hz (Natus Medical Incorporated, Pleasanton, CA, USA). Vestibular function was defined according to the angular vestibulo-ocular reflex (aVOR) gain, which was calculated using Otosuite 2.0 (Natus Medical Incorporated). Postoperative vHIT was performed on all patients in the CI-on condition.

The bithermal caloric reflex test was carried out according to the Fitzgerald–Hallpike technique, with the patient in a supine position, head flexed at 30° for 30 s, and auricular irrigation of 250 mL of water at 30° and 44 °C. The symmetry of the functionality was processed by the software Vestlab (Natus Medical Incorporated, Pleasanton, CA, USA) [12].

Written informed consent was obtained by each of the enrolled subjects and the study was carried out according to the 1964 Helsinki Declaration and its later amendments or comparable ethical standards.

## 3. Results

Resolution of vestibular symptoms was confirmed in all patients by normal scores obtained at the DHI questionnaire and by normal values as far as the reported quality of life was concerned. The audiological outcomes are reported in Table 1.

The vestibular test results, regarding the DHI questionnaire outcomes, are shown in Table 2, whereas the SF36-QOL results are reported in Table 3.

The PTA values and the speech recognition score (SRS) of all patients were comparable to those observed in our common implanted population; the only less satisfactory result concerned congenital deafness (Case 1).

VHIT showed normal or sub-normal gains of VOR after cochlear implantation for all patients and there was no significant difference between preoperative and postoperative outcomes (*p* > 0.05). The caloric test generally showed a partial hyporeflexia, often occurring with cold stimulation and usually bilaterally.

### 3.1. Case 1

A 44-year-old woman with congenital bilateral severe hearing loss of unknown origin, fitted with hearing aids until the age of 24, began to suffer from recurrent vertigo at the age of 42. The vertigo crises were rotatory with some episodes of the Tumarkin crisis, at least twice a week, with evident disabling consequences. The CT was normal, and the MRI performed with the hydrops protocol (3 Tesla MRI in 3D-fluid attenuated inversion recovery [FLAIR] sequences four hours after gadolinium injection) showed no enlargement of the vestibular labyrinth. DEH was finally diagnosed, a clinical entity correlated to MD, which is typically observed in patients who have been suffering from longstanding profound sensorineural hearing loss, no matter what its cause; the development of the disease is probably induced by delayed atrophy or fibrous obliteration of the endolymphatic resorptive system of the membranous labyrinth [13]. Nevertheless, given the bilateral nature of the congenital deafness, we were not able to identify the side affected by endolymphatic hydrops; therefore, in agreement with the patient, we decided to place a bilateral simultaneous cochlear implant. The surgery was performed without complications, with the complete insertion of the electrode arrays (Slim perimodiolar CI532 electrode array, Cochlear Ltd., Sydney, Australia, on both sides) through the round window.

No more vertigo crises were reported since the surgery, with a follow-up of two years. Concerning the hearing performance, the patient is still satisfied, although the speech recognition score (SRS) is not excellent (due to congenital deafness).

The patient reports a good quality of life and normal daily routines. The post-implantation vestibular tests were performed 13 months after surgery, with vestibular functionality preserved both at vHIT and caloric tests.

### 3.2. Case 2

A 61-year-old male with post-infectious right moderate hearing loss since adolescence developed contralateral delayed endolymphatic hydrops at the age of 55. The diagnosis was made upon the appearance of left tinnitus, documented left fluctuating hearing loss, and Lermoyez-type disabling vertiginous crises two/three times a week. Contrarily to the pathophysiology of ipsilateral DEH proposed in the previous case, the cause of the suffering of the second ear in contralateral DEH has not been explained yet: genetic mutations, inner ear malformations, or autoimmune diseases, even though no specific markers have been found yet, are possible theories at the basis of the disease [14]. In consideration of the bilateral hearing loss and the need to preserve residual hearing by avoiding destructive treatments on the vestibular system, we decided to place the CI on the left side (Flex 28 electrode array, Med-El, Innsbruck, Austria). Since that time, after 18 months of follow-up, the tinnitus and the disabling vertigo crises have disappeared. The patient successfully uses the CI on the left side and the hearing aid on the right side; the patient has a sporty lifestyle and feels confident even when hiking. The vestibular evaluations were performed 4 months after surgery.

### 3.3. Case 3

A 60-year-old woman developed bilateral hydropic symptoms with tinnitus, fluctuating hearing loss at the age of 36, and similar symptoms associated with vertigo after some years; two years before the CI, she developed two Tumarkin-type crises. We diagnosed a bilateral definite MD. The CT scan and the labyrinthine MRI were not significant. We could not practice any kind of labyrinthectomy because of the bilateral disease.

Since the hearing on the left side was severely compromised, and she wore the hearing aid on the right side, we treated the patient with a CI on the left side (Slim straight CI522 electrode array, Cochlear Ltd., Sydney, Australia). After surgery, the episodes of vertigo disappeared and the patient reported rare episodes of dizziness, only appearing when very tired. The vestibular evaluations were performed 9 months after surgery.

### 3.4. Case 4 and Case 5

Two patients, both diagnosed with bilateral definite MD, had severe bilateral hearing loss and typical disabling dizziness. The first (male, 58 years old) was implanted on the left side (Standard 31.5 mm electrode array, Med-El, Innsbruck, Austria) and was evaluated 6 years after surgery, whereas the second patient (female, 79 years old) was implanted on the right side (Slim straight CI522 electrode array, Cochlear Ltd., Sydney, Australia) and was evaluated one year after surgery. Both reported complete resolution of vertigo.

## 4. Discussion

The cases presented in this work have a common feature: all patients were implanted after a particular evolution of a hydropic syndrome or a defined MD, which led to severe hearing loss and disabling vestibular manifestations, with a bilateral involvement or the impossibility to identify the affected side for which a labyrinth deafferentation treatment was contraindicated. The purpose of the cochlear implantation was to restore auditory functioning; however, we were surprised by its positive vestibular effect. Indeed, all patients presented with an active and disabling vestibular syndrome before surgery, which has completely and permanently (so far) regressed after implant activation. Although a complete vestibular dysfunction is a well-known possible sequela after cochlear implantation, reported at rates ranging from about 15% to 70% in different studies [15], the postoperative vestibular evaluation of our patients never showed a complete absence of reflexes on the implanted side as might have been imagined given the resolution of vertigo, and a mild bilateral hyporeflexia was only revealed in some cases.

The vestibular function tests with VHIT carried out in the intercritical phases of MD, before surgery, had not shown any peripheral vestibular deficit, and such good outcomes, on the same side, were maintained even months or years after CI placement. Therefore, among all patients, the postoperative vHIT was generally normal (stimulation of type I cells, less involved in hydrops damage) whereas the caloric test was impaired (stimulation of type II cells, primarily damaged in MD). The reason for such a dissociation between these two vestibular test results must be sought among their intrinsic characteristics: the vHIT measures a stimulation at high frequencies (about 5 Hz) of type I cells, less involved in hydropic damage; on the contrary, the caloric test measures the stimulation at very low frequencies (about 0.002–0.004 Hz) of type II cells, mainly damaged in this type of disease [16]. A dissociation between caloric testing and vHIT has been described in cases with endolymphatic hydrops due to altered mechanics of the inner ear [17] and has been described as a distinctive pattern of a vestibular deficit in delayed endolymphatic hydrops [18].

Furthermore, some studies in the literature claim that caloric testing is more sensitive for non-acute vestibular dysfunction than vHIT [16]: a recent update on vestibular diagnosis by Starkov shows that when vestibular hypofunction is suspected, it might be recommended to start with vHIT due to its low burden for the tested subject. If vHIT results are abnormal, no other vestibular testing is necessary. However, in the case of normal vHIT results, performing caloric testing might be advisable, since caloric testing seems to be more sensitive than vHIT in detecting vestibular hypofunction, especially in MD [17].

According to Rah et al., a mean 10°/s reduction in the angular slow phase velocity (ASPV), which is a quantitative variable used to determine vestibular hypofunction, was seen in the implanted ear after CI with caloric testing [19]; similarly, Abramides et al. reported a 20% impairment at caloric testing after CI [20].

The second patient was an interesting case for two reasons: the first is related to the quite uncommon pathology, contralateral delayed endolymphatic hydrops: unfortunately, he never responded to conservative medical treatments, as seen for example in three similar cases reported by Reynard et al. [21]. Another interesting aspect of this case was the deep psychological involvement, which led the patient to reduce his general daily commitment and to fill out a docket with each small manifestation of the disease in detail. The psychological implications of MD were investigated in a review by Kirby et al., where important components of anxiety and post-traumatic stress disease (PTSD) were demonstrated, and psychological support was suggested for these patients [22].

Auditory results were satisfactory in all cases, although the first patient did not obtain a good SRS without lip reading. This finding is consistent with what has been observed in sound-deprived patients but does not contraindicate the CI, especially in case the patient was correctly stimulated with hearing aids and had good lip-reading skills [23].

As for the literature, most of the articles on cochlear implantation in MD deal only with the auditory effects of CI, and very few authors have focused on its vestibular effects.

McRackan et al. [24] evaluated 21 implanted patients with definite MD: 90% had bilateral disease and 28.6% (6 patients) had active vestibular symptoms; after cochlear implantation, 5 patients reported a reduction in vestibular disease, 2 patients were able to discontinue medication, and only 1 patient reported no benefit from CI. Vestibular evaluations and questionnaires were not carried out. In terms of hearing, they reported worse results than the general implanted population, and better results were seen in patients with long-standing MD, assuming more stable nerve conduction.

Mukherjee et al. [25] presented a series of CI patients suffering from MD, with or without labyrinthectomy, exploring vertigo and hearing loss. All patients implanted in association with labyrinthectomy clearly reported the disappearance of vertigo. The 22 patients who placed CI alone had a silent vestibular disease, but half of them reported persistence of some vestibular manifestations, such as benign paroxysmal positional vertigo (BPPV) or dizziness after surgery; no further vestibular test was performed. Furthermore, nine cases of MD submitted to CI in the suffering ear were presented in a work by Lustig et al. [26]. Two patients reported the complete resolution of the vestibular manifestations, disabled until surgery, but once again, no vestibular evaluations were performed. Outside the context of the MD, the effect of the CI on the vestibular system was investigated in a few studies. In a 2015 publication, it was reported that electrode insertion can compromise vestibular function, as a reduction in the gain of the vestibulo-ocular reflex (VOR) was highlighted at vHIT, more common and more consistent in the case of electrode insertion in the vestibular scale of the cochlea [27].

Potential reasons for the resolution of vestibular symptoms after CI positioning may include an alteration in the inner ear fluid homeostasis or an inflammatory state with consequent fibrosis due to the presence of the electrode itself, which might alter the endolymphatic flow in the semicircular canal. The study conducted on guinea pigs by Smeds et al. confirmed these changes in the outflow of the endolymphatic system, showing both morphological evidence on micro-CT and electrocochleography responses consistent with the presence of secondary endolymphatic hydrops (SEH) in the first few weeks after CI, due to the increased volume of scala media causing ballooning of the compliant Reissner’s membrane and downward displacement of the basilar membrane [28]. Similarly, Ferster et al., in their review, reported an incidence of SEH between 42% and 59% after cochlear implantation [29]. Anatomically, due to the proximity of the auditory and vestibular structures, cochlear implantation could result in activation of the vestibular afferents since the current spreads through the fluid-filled ductus reuniens [30]. Moreover, the saccule, which is connected to the cochlea via the ductus reuniens, is nearest to the basal turn of the cochlea where damage from the electrode insertion may most likely occur. Su-Velez, after examining the histopathology of human temporal bones with cochlear endolymphatic hydrops after CI, demonstrated the onset of cochlear hydrops in 88% of the saccules secondary to fibrosis of the ductus reuniens [15].

Another possibility to explain the resolution of the hydropic vestibular symptoms after CI positioning is the intraoperative perilymph loss due to opening the round window, leading to a reduction in scala tympani pressure and, therefore, causing the basilar membrane to be displaced downwards; however, such hypothesis does not explain the persistence of good long-term results, when the cochlea is (presumably) sealed. On the contrary, it is unlikely that such results could be related to direct surgical trauma as we have never performed concomitant vestibular labyrinthectomy and the insertion of the electrode has always been atraumatic, as shown by the values in the range at vHIT, and through a round-window approach. In this regard, Frodlund et al. showed how the cochleostomy approach and the use of straight electrodes can cause intracochlear trauma associated with the development of fibrosis of the ductus reuniens and, therefore, with alterations of endolymphatic homeostasis [31]. Moreover, as mentioned by Green et al., around 70% of the vestibular symptoms associated with MD vanish over the long term; thus, the vestibular crisis disappearances after CIs in patients with disabling MD may also be considered as part of the ongoing disease itself [32].

There is also evidence of improved gait and balance stability after cochlear implantation. Authors reported that unilateral CI could alter a previously uncompensated vestibular injury; thus, inducing compensation, greater self-confidence, and improvement in spatial orientation [33,34]. In a recent publication, gait stability was evaluated in 21 patients after CI, finding better parameters than those observed before surgery; the authors report that videonystagmography, vHIT, and VEMP were performed to demonstrate these data, but results are not described in the study; furthermore, these patients did not present vestibular symptoms before implantation [35].

Finally, even though it is an uncompensated disabling MD, the placebo effect of the intervention should not be underestimated as a possible cause of symptom improvement. In fact, the influences of psychological factors in MD and the interactions that exist between anxiety and the resurgence of vertigo crises are commonly known [36]. On this topic, a Cochrane systematic review of the literature on surgery for MD found no significant differences in symptom relief between the placebo group (either simple mastoidectomy or ventilation tube placement) and the treatment group with destructive surgery, even after nine years of follow-up [37].

The present study has some limitations: the small number of patients included is a critical point but is linked to relatively rare clinical conditions reported (SEH, bilateral MD, no labyrinthectomy). The second limitation is the low number of vestibular evaluations performed prior to surgery and the lack of early postoperative assessments as only a few vestibular tests were performed a couple of months after CI activation.

Due to the retrospective nature of the study, a thorough preoperative vestibular evaluation is not available since the aim of the surgery was primarily to restore hearing and such complete resolution of vestibular symptoms was not predictable prior to CI placement. Furthermore, the diagnosis of MD is only clinical (apart from a pure-tone audiometry, which is essential) and, according to the Classification Committee of the Bárány Society, the execution of multiple vestibular instrumental assessments, such as videonystagmography, caloric tests, or labyrinthine MRIs, is not required among the diagnostic criteria of definite MD [1].

Certainly, a preoperative caloric test, an electrocochleography, and a skull vibration-induced nystagmus test (SVIN) could have helped in identifying the responsible ear. Indeed, the SVIN, which is positive in 28–71% of definite MD and often results in irritation in the pre-crisis period, is a simple test suggested in the immediate postoperative period as it could reveal induced nystagmus and, therefore, early variation in the vestibular function [38]. Moreover, the integration of all these tests, together with a labyrinthine MRI performed according to the 3D-FLAIR protocol and late sequences after gadolinium injection could be particularly interesting in case of severe hearing loss [39,40] and could provide excellent possibilities to reveal the pathological side and to understand the effects of CI on the vestibular system as well. None of the patients with definite MD or delayed endolymphatic hydrops included in this study were diagnosed on the basis of the preoperative MRI, which was normal in all cases.

## 5. Conclusions

Although we are not recommending the use of CI as the primary surgical treatment for definite MD, the results obtained from this study may prove useful in the therapeutic counseling of patients with uncompensated definite MD, unresponsive to common medical treatments. In fact, cochlear implantation has proved its efficacy—not only in restoring hearing and reducing the tinnitus but also in controlling disabling vestibular manifestations in patients with profound deafness and vertigo crises with hydropic characteristics.

The real advantage of this choice is the possibility of preserving the vestibule in bilateral disease, especially in the case of a previous contralateral labyrinthectomy or regarding the impossibility of identifying the responsible side; moreover, the solution of a bilateral CI must be kept in mind in case of bilateral MD.

The clinical cases presented are quite surprising and encouraging, as some similar findings begin to emerge from the literature. However, further considerations are needed, and some questions are pending: Is the opening of the cochlea and the subsequent insertion of electrodes, or electromagnetic stimulation, responsible for the modification of the vestibular function? How important is auditory restoration for vestibular function? To what extent could the resolution of vertigo be attributable to the natural evolution of a definite and long-standing MD? Only an increase in the number of clinical cases and a complete (and systematic) vestibular evaluation during the whole diagnostic and therapeutic process could likely lead us to solve these questions.

## Figures and Tables

**Table 1:** Auditory outcomes before and after cochlear implant placement.

	Pre-Implant PTA (0.5–1–2–3 kHz)	Post-Implant PTA (0.5–1–2–3 kHz)	Pre-Implant SRS	Post-Implant SRS
Case 1	Left: 110 dB Right: 112 dB	Left: 22 dB Right: 22 dB	0%	20% at 60 dB
Case 2	Left: 92 dB Right: 70 dB	Right (CI alone): 27 dB	Left: 20% at 80 dB Right: 90% at 80 dB	100% at 60 dB
Case 3	Left: 115 dB Right: 102 dB	Left (CI alone): 22 dB	Left: 0% at 80 dB Right: 20% at 80 dB	100% at 60 dB
Case 4	Left: 105 dB Right: 100 dB	Left (CI alone): 30 dB	Left: 0% Right: 20% at 80 dB	80% at 60 dB
Case 5	Left: 110 dB Right: 110 dB	Right (CI alone): 25 dB	0%	80% at 60 dB

PTA: pure-tone average. SRS: speech recognition score. Pre-implant evaluations were performed with headphones; post-implant evaluations were performed in free-field and quiet conditions. Percentages refer to the maximal intelligibilities reached.

**Table 2:** Vestibular outcomes before and after cochlear implant placement.

	Pre-Implant vHIT	Pre-Implant DHI	Post-Implant vHIT	Post-Implant Caloric Test	Post-Implant DHI
Case 1	LH 0.93 ± 0.12; RH 0.98 ± 0.13	56	LH 0.89 ± 0.24; RH 0.53 ± 0.45	WR 13.3 WL −13.2	14
	LA 0.93 ± 0.12; RP 0.81 ± 0.07		LA 0.61 ± 0.24; RP 0.54 ± 0.13	CR −3.1 CL 2.6	
	RA 0.66 ± 0.36; LP 0.77 ± 0.08 ^+^		RA 0.70 ± 0.15; LP 0.54 ± 0.13	VP 1.7% DP −0.9% ^§^	
Case 2	LH 0.98 ± 0.25; RH 0.92 ± 0.18	28	LH 0.93 ± 0.04; RH 0.97 ± 0.02	WR 7.2 WL −6.9	0
	LA 0.83 ± 0.13; RP 0.78 ± 0.08		LA 0.72 ± 0.11; RP 0.60 ± 0.12	CR −7.6 CL 5.4	
	RA 1.25 ± 0.27; LP 1.06 ± 0.10		RA 1.27 ± 0.11; LP 1.13 ± 0.05	VP 9.1% DP −6.6%	
Case 3	LH 0.90 ± 0.16; RH 1.02 ± 0.09	42	LH 0.84 ± 0.06; RH 0.93 ± 0.09	WR 4.6 WL −5.5	10
	LA 0.82 ± 0.08; RP 0.95 ± 0.11		LA 0.76 ± 0.21; RP 0.84 ± 0.15	CR −8.0 CL 4.6	
	RA 0.88 ± 0.28; LP 0.94 ± 0.12		RA 0.80 ± 0.18; LP 0.85 ± 0.07	VP 10.7% DP −18.5%	
Case 4	LH 1.04 ± 0.14; RH 0.85 ± 0.28	34	LH 0.85 ± 0.14; RH 0.74 ± 0.29	WR 5.0 WL −3.6	10
	LA 0.86 ± 0.18; RP 1.06 ± 0.14		LA 0.92 ± 0.40; RP 1.32 ± 0.09	CR −6.5 CL 3.7	
	RA 1.22 ± 0.14; LP 0.65 ± 0.08		RA 1.28 ± 0.31; LP 0.50 ± 0.16	VP 22.3% DP −7.8%	
Case 5	LH 0.96 ± 0.04; RH 0.91 ± 0.05	38	LH 0.94 ± 0.08; RH 0.96 ± 0.09	WR 5.9 WL −2.9	14
	LA 1.02 ± 0.15; RP 0.93 ± 0.09		LA 0.89 ± 0.33; RP 0.79 ± 0.08	CR −4.9 CL 2.3	
	RA 0.89± 0.29; LP 0.98 ± 0.17		RA 0.93 ± 0.38; LP 0.88 ± 0.13	VP 34.8% DP 2.7%	

^+^ LH: left horizontal, RH: right horizontal, LA: left anterior, RP: right posterior, RA: right anterior, LP: left posterior. Measures expressing the ratio between the velocity of the movement of the eye and the velocity of the movement of the head are expressed in a medium ± standard deviation of 10 tests for each semicircular channel. ^§^ Measures expressed in °/s of the slow phase velocity (SPV) of the nystagmus. WR: warm right; WL: warm left; CR: cold right; CL: cold left; VP: vestibular paresis or unilateral weakness; DP: directional preponderance.

**Table 3:** Outcomes of the SF-36 QOL questionnaire.

	Physical Functioning	Limitations Due to Physical Health	Limitations Due to Emotional Problems	Energy/ Fatigue	Emotional Well-Being	Social Functioning	Pain	General Health
Case 1	95	100	100	35	56	64	100	15
Case 2	100	100	100	100	96	100	100	84
Case 3	100	100	100	65	80	90	90	88
Case 4	100	100	100	65	80	100	100	84
Case 5	95	95	100	55	80	80	100	88
